# The Identification of New Pharmacological Targets for the Treatment of Glaucoma: A Network Pharmacology Approach

**DOI:** 10.3390/ph17101333

**Published:** 2024-10-05

**Authors:** Erika Giuffrida, Chiara Bianca Maria Platania, Francesca Lazzara, Federica Conti, Nicoletta Marcantonio, Filippo Drago, Claudio Bucolo

**Affiliations:** 1 Department of Biomedical and Biotechnological Sciences, School of Medicine, University of Catania, 95125 Catania, Italy; erika.giuffri@gmail.com (E.G.); chiara.platania@unict.it (C.B.M.P.); francesca.lazzara@unict.it (F.L.); federica.conti@unict.it (F.C.); nicolettamarcantonio61@gmail.com (N.M.); fdrago@unict.it (F.D.); 2 Center for Research in Ocular Pharmacology-CERFO, University of Catania, 95125 Catania, Italy

**Keywords:** network pharmacology, computational systems biology, pharmacological targets, neuroinflammation, glaucoma

## Abstract

**Background:** Glaucoma is a progressive optic neuropathy characterized by the neurodegeneration and death of retinal ganglion cells (RGCs), leading to blindness. Current glaucoma interventions reduce intraocular pressure but do not address retinal neurodegeneration. In this effort, to identify new pharmacological targets for glaucoma management, we employed a network pharmacology approach. **Methods:** We first retrieved transcriptomic data from GEO, an NCBI database, and carried out GEO2R (an interactive web tool aimed at comparing two or more groups of samples in a GEO dataset). The GEO2R statistical analysis aimed at identifying the top differentially expressed genes (DEGs) and used these as input of STRING (Search Tool for the Retrieval of Interacting Genes/Proteins) app within Cytoscape software, which builds networks of proteins starting from input DEGs. Analyses of centrality metrics using Cytoscape were carried out to identify nodes (genes or proteins) involved in network stability. We also employed the web-server software MIRNET 2.0 to build miRNA–target interaction networks for a re-analysis of the GSE105269 dataset, which reports analyses of microRNA expressions. **Results:** The pharmacological targets, identified in silico through analyses of the centrality metrics carried out with Cytoscape, were rescored based on correlations with entries in the PubMed and clinicaltrials.gov databases. When there was no match (82 out of 135 identified central nodes, in 8 analyzed networks), targets were considered “potential innovative” targets for the treatment of glaucoma, after further validation studies. **Conclusions:** Several druggable targets, such as GPCRs (e.g., 5-hydroxytryptamine 5A (5-HT5A) and adenosine A_2B_ receptors) and enzymes (e.g., lactate dehydrogenase A or monoamine oxidase B), were found to be rescored as “potential innovative” pharmacological targets for glaucoma treatment.

## 1. Introduction

Glaucoma is a progressive optic neuropathy and one of the leading causes of irreversible blindness worldwide. In 2020, 3.6 million people, aged 50 years or older, irreversibly lost their vision due to glaucoma, which accounted for a total of 11% of global blindness among adults of this age [1]. Furthermore, an increased burden of care in glaucoma management is estimated due to the ageing of populations worldwide, especially in the industrialized world [2].

The death of retinal ganglion cells (RGCs), optic disc cupping, and alterations of the peripheral and central visual fields represent the hallmarks of this disease [3,4]. Although glaucoma etiology has not yet been fully elucidated, the loss of RGCs, along with a damage of the optic nerve head (ONH), is linked to multifactorial pathogenic mechanisms such as axonal transport blockades, the deprivation of neurotrophic factors, mitochondrial dysfunction, excitotoxic damage, oxidative stress, and reactive gliosis [5]. Although several risk factors for glaucoma have been identified, such as aging, ethnic origin, and family history, as well as polygenic associations, high intraocular pressure (IOP, >21 mmHg) remains the main and the only modifiable risk factor of glaucoma [6,7]. The increase of IOP is caused by an imbalance between the production and the drainage of the aqueous humor (AH). Up to now, approved topical ocular hypotensive drugs belong to the following therapeutic classes: prostaglandin derivatives, beta-blockers, carbonic anhydrase inhibitors, sympathomimetics, miotics, and the recently approved rho-kinase inhibitors [8]. Prostaglandin analogues and beta-blockers represent first- and second-line treatments, respectively, followed by carbonic anhydrase inhibitors and alpha-2 adrenergic agonists [9]. In the case of an inadequate response to these drugs, ocular surgery, such as trabeculectomy, is recommended. It should be mentioned that normotensive glaucoma patients may take advantage of IOP-lowering drugs, as reported in a clinical trial where a decreased odds ratio for disease progression was registered in patients with glaucoma without hypertension [10]. However, about 40% of patients do not respond to IOP-lowering drugs, and in such patients the disease still slowly progresses to sight loss [11]. Therefore, new therapeutic approaches, able to preserve the retinal visual system and to directly protect RGCs, are needed [12].

In this regard, network pharmacology can be used to identify, in silico, new pharmacological targets for the treatment of complex and multifactorial diseases, including Alzheimer’s disease [13], COVID-19 infections [14], and diabetic retinopathy [15], and for the management of cancer metastasis [16]. Through the application of this method, a disease can be represented as a network, in which the nodes (genes, proteins, molecules, and pathways), establishing multiple interactions (edges), provide a holistic representation of the pathology [17]. Moreover, the integration of multi-layer omics data would help to unveil the pathogenic mechanisms of predicted new diseases [17,18].

Our in silico study exploited the network pharmacology approach, which borrows methods from the in silico systems biology approach, also defined as the reverse engineering approach [19]. Specifically, we hereby re-analyzed experimental data already published or deposited in databases to build a network with new nodes and connections through the enrichment of knowledge approaches [15]. A network centrality analysis then helped with the identification of the nodes essential for network stability [20,21], which can be considered predicted pharmacological targets [15] for the management of a given disease. The following steps were applied, according to our previously published work [15]: first, we carried out a re-analysis of transcriptomic data retrieved from the Gene Expression Omnibus (GEO) database [22] of expression datasets associated with tissues from glaucoma patients and control subjects of experimental in vivo models of glaucoma. The datasets were directly analyzed using the GEO2R statistical tool, which was employed to identify the genes that were significantly (*p* < 0.05) differentially expressed (DEGs) (i.e., glaucoma vs. healthy control) [23,24]. Thereafter, we applied the following reverse engineering approach, giving, as input, the DEGs from the GEO2R analysis to the STRING (Search Tool for the Retrieval of Interacting Genes/Proteins) app embedded within the Cytoscape software v. 3.7.0 [25] (https://apps.cytoscape.org/apps/stringapp, accessed on 1 March 2022). The STRING app ensures enrichment of the information it receives from enrichment analyses of gene ontology, KEGG, Reactome, and Wiki Pathways. Finally, the predicted key genes, identified after a centrality analysis of the networks, were rescored as predicted new or not new, on the basis of the lack or presence of a matching with entries on the PubMed and clinicaltrials.gov databases, respectively.

The network pharmacology re-analysis evidenced connections with pathways already experimentally associated with glaucoma pathogenesis, e.g., inflammatory pathways and pathways involved in the regulation of the immune system and fibrosis [26,27]. Furthermore, our study evidenced that several G protein-coupled receptors (GPCRs), ion channels, or enzymes could be involved in the pathological mechanisms of glaucoma. In particular, metabotropic serotoninergic and adenosine receptors, such as the 5-hydroxytryptamine 5A (5-HT5A) and adenosine A_2B_ receptors, were identified as innovative (not yet investigated) drug targets for glaucoma treatment. Moreover, different ligand–gated ion channels belonging to purinergic (e.g., the purinergic ionotropic receptor P2X3) and glutamatergic systems (e.g., the glutamate ionotropic receptor AMPA type subunit 1), might be predicted new pharmacological targets for glaucoma treatment, since no in vitro, in vivo, or clinical studies have been linked to these targets yet. Finally, a series of enzymes, such as lactate dehydrogenase A or monoamine oxidase B, were also identified as predicted new targets for the management of glaucoma.

## 2. Results

The search in the GEO database for GEO datasets associated with glaucoma (search query “glaucoma”) resulted in six GEO datasets (Table 1) reporting transcriptomic data associated with preclinical in vivo models of glaucoma and postmortem isolated human eye tissues. These retrieved GEO datasets were automatically linked to the GEO2R analysis tool.

### 2.1. GSE133563: Analysis of Retinal Transcriptomic Data 2 Weeks after Optic Nerve Crush

**Figure 1:**
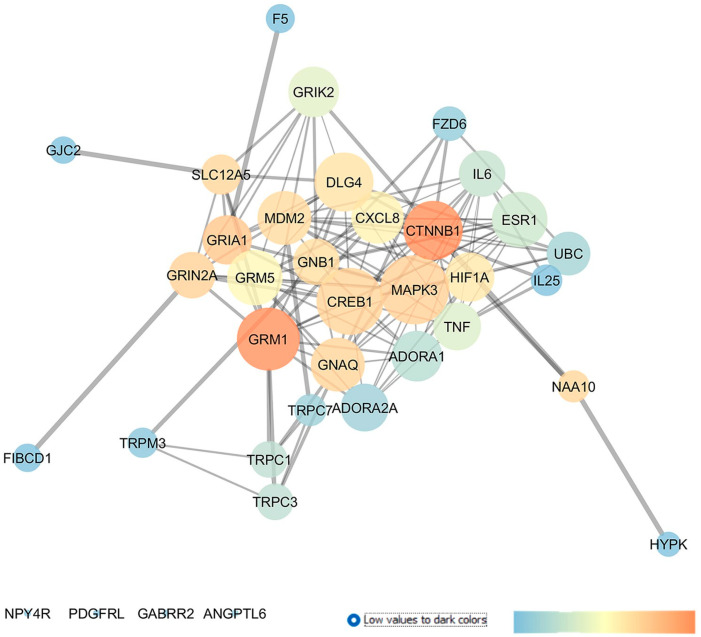
Dysregulated genes in retina of Lister Hooded rats 2 weeks after an optic nerve crush (ONC) injury. Network was analyzed with Cytoscape. Nodes are represented on the basis of closeness centrality values (node dimension) and betweenness centrality values, as shown by the legend of this figure (temperature color scale blue < red). Edge thickness is proportional to edge betweenness values.

GSE133563 is a repository of transcriptomes of the retinas of Lister Hooded rats (aged 8 weeks) 14 days after an optic nerve crush (ONC) compared to sham-operated animals, i.e., the control (CTRL). Since the ONC caused a strong reduction in the number of RGCs, this transcriptome analysis provided information about adaptive modifications in response to the loss of RGCs [28]. In this regard, these authors found that the complement cascade and Notch signaling pathways were the main dysregulated pathways in RGCs after an ONC injury. Moreover, oxidative stress and KIT receptor pathways were also highlighted as enriched pathways. In line with these findings, our re-analysis evidenced the involvement of different genes related to oxidative stress and inflammatory pathways in ONC-induced damages on retinal tissues. Starting from the 250 DEGs, retrieved through a GEO2R analysis of GSE133563, we have built an enriched network, as shown in Figure 1. From a centrality analysis of this network and from rescoring, 12 out of 23 identified genes were predicted as new pharmacological targets for the treatment of glaucoma, since no entries, related to literature studies or clinical trials, were retrieved: GRIA1 (glutamate ionotropic receptor AMPA type subunit 1), GRIK2 (glutamate ionotropic receptor kainate type subunit 2), TRPC3 (transient receptor potential cation channel subfamily C member 3), TRPC7 (transient receptor potential cation channel subfamily C member 7), HYPK (huntingtin interacting protein K), FIBCD1 (fibrinogen C domain containing 1), GJC2 (gap junction protein gamma 2), FZD6 (frizzled class receptor 6), GABRR2 (gamma-aminobutyric acid type A receptor subunit rho2), ANGPTL6 (angiopoietin like 6), IL25 (interleukin 25), and F5 (coagulation factor V).

Moreover, this network analysis evidenced 11 genes already experimentally linked to glaucoma pathogenesis. Among these, the glutamate metabotropic receptors mGluR1 and mGluR5, along with the adenosine A_1_ and A_2A_ receptors, showed elevated centrality values in the network. Other interesting druggable targets were two transient receptor potential cation channels, encoded by TRPC1, found upregulated in glaucoma lamina cribrosa cells [33] and TRPM3. Additionally, DLG4 (discs large MAGUK scaffold protein 4) and several proinflammatory chemokines and cytokines already known to have a role in glaucoma, e.g., C-X-C motif chemokine ligand 8 (CXCL8), tumor necrosis factor (TNF), and interleukin-6 (IL-6), were other central nodes in the network. Specifically, IL-6 was found to be upregulated in the tears of POAG patients [34] and in the aqueous humor of angle-closure glaucoma (ACG) patients, respectively [35]. Moreover, a clinical study evaluated the effect of latanoprost (IOP-lowering drug) on IL-6 levels in the tear films of recruited subjects (NCT04957329). Finally, HIF1A (hypoxia inducible factor 1 subunit alpha), a transcription factor activated in tissues under hypoxic conditions and a regulator of several downstream pathways, i.e., of the vascular endothelial growth factor (VEGF), displayed high closeness and betweenness centrality values in the network. Interestingly, HIF-1α was found to be upregulated in tissues from glaucoma patients [36] and in experimental animal models of glaucoma, such as in mice [37] and rats [38].

### 2.2. GSE105269: Analysis of Differentially Expressed miRNAs in Aqueous Humor of Exfoliation Glaucoma and Primary Open-Angle Glaucoma Patients

GSE105269 is a collection of microRNAs (miRNAs) expression profile in AH of patients with exfoliation glaucoma (XFG), primary open-angle glaucoma (POAG) and cataract controls (CTRLs). miRNAs are small single-stranded noncoding RNAs that regulate gene expression at post-transcriptional level, through inhibition of the translation of messenger RNAs (mRNAs) or by promoting mRNA degradation [39]. As suggested by Drewry et al., 2018 [29], that deposited the GSE105269, miRNAs in AH could not only have key roles in disease onset progression but also in AH outflow dysregulation. Therefore, we exploited these data to identify new genes, that are also targets of differentially expressed (DE) miRNAs in GSE105269. At first, a GEO2R analysis was carried out to identify the most significant DE miRNAs in each group comparison, i.e., POAG vs. CTRL and XFG vs. CTRL. After this, the top 30 DE miRNAs of the GEO2R analysis (listed in the Supplementary Materials, List S1, S2) were set as an input of the MIRNET web server to create a miRNA–target gene network that was then exported and analyzed with the Cytoscape environment.

#### 2.2.1. GSE105269 AH from Primary Open-Angle Glaucoma Patients

**Figure 2:**
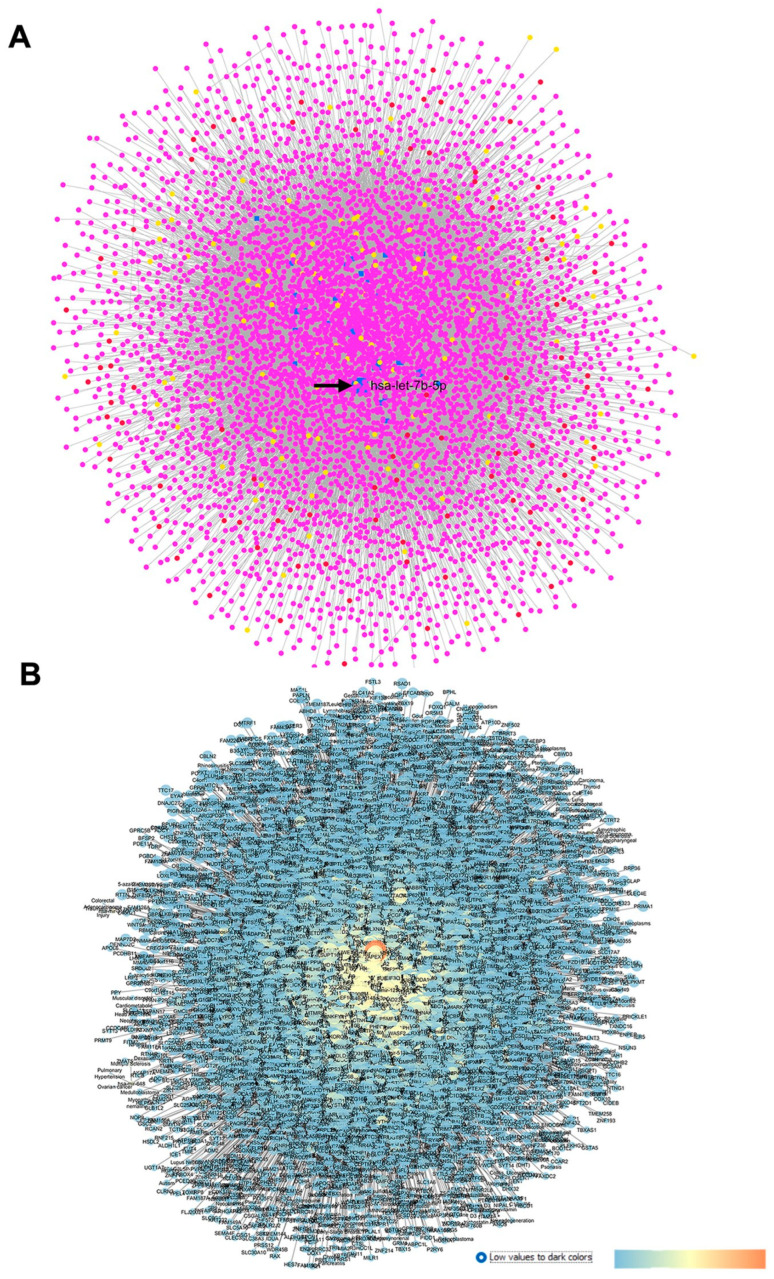
GSE105269 network: primary open-angle vs. CTRL cataract patients. (**A**) Output of MIRNET network within the MIRNET environment. Magenta nodes correspond to genes, blue nodes to miRNAs, yellow nodes to compounds (predicted to be able to modulate miRNA expressions), and red nodes to diseases associated to miRNAs. The arrow indicates the has-let-7b-5p miRNA, i.e., the most central node of this network. (**B**) For the MIRNET-generated network represented within Cytoscape, centrality parameters are mapped as follows: closeness centrality is proportional to node dimension; betweenness centrality is represented with a temperature color scale (blue to red for increasing values, as shown by the legend of this figure); and edge betweenness is proportional to edge thickness.

After miRNA profiling, Drewry et al. (2018) [29] identified three significant DE miRNAs in the POAG group compared to the CTRL group: hsa-miR-125b-5p, hsa-miR-302d-3p, and hsa-miR-451a. Furthermore, by evaluating the target genes of the above-mentioned DE miRNAs, genes related to the AKT and Bcl-2-regulated apoptosis signaling pathways were identified as gene targets of these miRNAs [29]. Our GEO2R analysis was carried out by comparing 12 AH samples of the POAG group with the 11 samples of the CTRL group. This analysis evidenced that hsa-miR-517c-3p, hsa-miR-339-5p, and hsa-miR-375 were the most significant DE miRNAs, and only hsa-miR-375 was previously found to be upregulated in the AH of patients with normal-tension glaucoma (NTG) compared to control subjects [40]. After the GEO2R analysis, the top 30 DE miRNAs were set as an input of the MIRNET web server, and a network was generated on the basis of miRNAs and their target genes. In the MIRNET network, shown in Figure 2A, the miRNAs (nodes) with the highest degree of centrality were hsa-let-7b-5p, hsa-mir-484, and hsa-mir-21-5p. For these miRNAs, no hits were retrieved on Pubmed. Furthermore, the above-mentioned hsa-mir-375, hsa-mir-125b-5p (one of the DE identified in Drewry et al. (2018) [29], and hsa-mir-302b-3p displayed high centrality values in our network. Meanwhile, the genes showing the highest degree and betweenness centrality values were HNF4A (hepatocyte nuclear factor 4 alpha), APP (amyloid beta precursor protein), and SUMO2 (small ubiquitin-like modifier 2).

An enrichment analysis of the Kyoto encyclopedia of genes and genomes (KEGG), aimed at linking genomic data with higher-order functional information [41], was carried out with MIRNET, which evidenced the following signaling pathways known to be implicated in this disease: the “Neurotrophin signaling pathway” (linked nodes: GRB2, YWHAZ, YWHAB, AKT1, and BAD); the “Insulin signaling pathway” (linked nodes: PYGB, FLOT1, and GRB2); the “p53 signaling pathway”; “Apoptosis”; and “Axon guidance”. Pathways such as “Cholinergic synapse” were enriched by genes encoding CHRM3 and CHRNA6. Moreover, “Dopaminergic synapse” (linked nodes: GRI2A, GRIN2B, and GRIA4) and “Long-term potentiation” could be also implicated in this disease.

Then, the MIRNET network, providing miRNA–target genes interactions, was exported in Cytoscape and represented according to centrality metric parameters (Figure 2B).

Overall, after this network analysis, we found that 8 genes, out of 17, encode new pharmacological targets, which have not yet been associated with glaucoma and were, thereby, rescored as innovative targets: ADORA2B (the adenosine A_2B_ receptor); HTR1D (the 5-hydroxytryptamine 1D receptor, 5-HT1D); P2RX4 (the purinergic receptor P2X4); HSPA8 (heat shock protein family A (Hsp70) member 8); TNRC6A (the trinucleotide repeat-containing adaptor 6A); NUFIP2 (nuclear FMR1-interacting protein 2); SRRM2 (serine/arginine repetitive matrix 2, required for pre-mRNA splicing as a component of the spliceosome); and SUMO2 (small ubiquitin-like modifier 2).

Within the miRNA–gene network, built on the basis of a re-analysis of GSE105269 POAG vs. CTRL patients, ELAVL1 (embryonic lethal abnormal vision-like protein 1) was the most central gene node of this network (Table 2). ELAVL1 encodes an RNA-binding protein which is able to stabilize the mRNAs, with an AU-rich element, of several genes, such as growth factors and inflammatory cytokines. ELAVL1 has already been proven to be involved in glaucoma pathogenesis [42,43]. The second most central node was APP (the amyloid beta precursor protein), implicated in patients with Alzheimer’s disease (AD) and also in glaucomatous neurodegeneration [44,45]. Moreover, EGR1 (early growth response 1), IGF1R (the insulin-like growth factor 1 receptor), THBS1 (thrombospondin 1), CDKN1A (the cyclin-dependent kinase inhibitor 1A), P2RY2 (the purinergic receptor P2Y2), ADRA2A (the alpha-2A adrenergic receptor and α2A adrenoceptor), and ADRB2 (the beta-2 adrenergic receptor) were other genes displaying elevated closeness centrality values in the network. EGR1 was found to be significantly upregulated in C57BL/6 mice, following an axonal injury through RNA-sequencing technology [46]. The insulin-like growth factor 1 receptor (IGF1R) was found to be phosphorylated in rat RGCs shortly after an episcleral vein cauterization (EVC), identifying this activation as a compensatory response to EVC stress [47]. Finally, a missense variant of the THBS1 gene was associated with POAG, as reported in a study that carried whole-genome sequencing (WGS) in a large cohort of POAG subjects with an elevated IOP [48]. With respect to the cyclin-dependent kinase inhibitor 1A, this encoding gene was associated with an increased risk of POAG, as reported in a meta-analysis by Springelkamp et al. (2017) [49]. The metabotropic purinergic receptor P2Y2 has already been investigated as a modulator of IOPs in vivo [50,51]. With respect to adrenergic receptors, the alpha-2A and beta-2 adrenoceptors are the target of two IOP-lowering drug classes, i.e., alpha-2adrenergic-receptor agonists and beta-blockers. In this regard, an interventional study (NCT00279253) assessed the effects of the intravenous administration of clonidine (an alpha-2 adrenergic agonist) on the ocular blood flow and IOP of healthy subjects; however, no report on this study has been published yet. Moreover, several studies regarding the association of ADRB2 genetic polymorphisms with POAG were retrieved from the PubMed database [52,53,54]. Specifically, ADRB2 polymorphisms were associated with glaucoma in an Indian population [52], and in Japanese patients [54] and were also found to correlate to increased therapeutical response to β-blockers eye drops, used for the treatment of ocular hypertension [55].

#### 2.2.2. GSE105269 AH of Exfoliation Glaucoma

**Figure 3:**
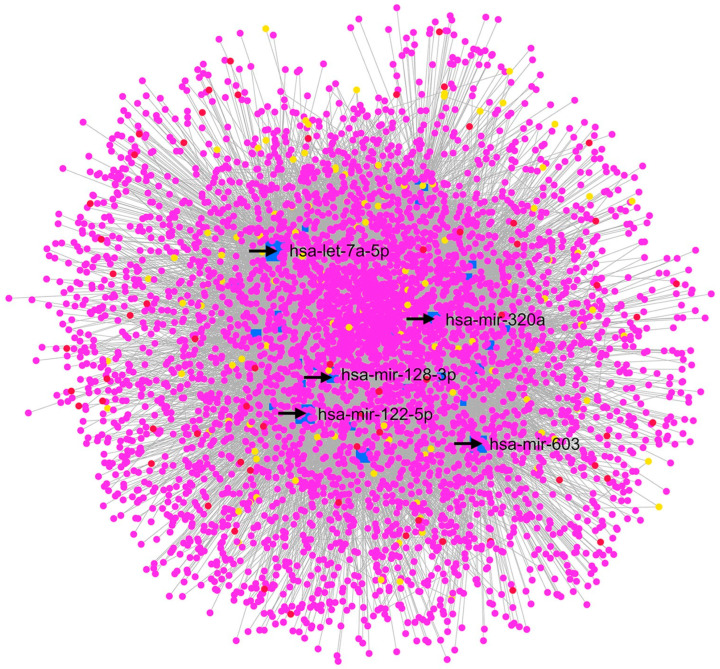
GSE105269 MIRNET network, XFG vs. CTRL. The MIRNET web server provided miRNA–gene, miRNA–disease, and miRNA–small molecule interactions, depicted in this network. In this network, has-let-7a-5p, hsa-miR-122-5p, hsa-miR-320a, hsa-miR-128-3p, and hsa-miR-603 are the nodes with the highest centrality values. Magenta nodes correspond to genes, blue nodes to miRNAs, yellow nodes to compounds (predicted to be able to modulate miRNA expressions), and red nodes to diseases associated with miRNAs.

XFG is the most common secondary form of POAG, a complication of the exfoliation syndrome (XFS), characterized by the accumulation of fibrillar extracellular material in the anterior segment of the eye, leading to the obstruction of AH outflow pathways and an increase in the IOP [56]. Drewry et al. (2018) [29] reported five miRNAs with significantly different expressions in an XFG group compared to a CTRL group: hsa-miR-122-5p, hsa-miR-3144-3p, hsa-miR-320a, hsa-miR-320e, and hsa-miR-630. Moreover, these authors underlined BCL2L2, IGF1R, and RAC1 as the genes targeted by most of the identified DE miRNAs in the XFG group. Our GEO2R analysis revealed that the most significant DE miRNAs from samples of the XFG and CTRL groups were hsa-miR-548ar-5p, hsa-miR-612, hsa-miR-122-5p, and hsa-miR-320a. The hsa-miR-122-5p and hsa-miR-320a were validated by Drewry et al. (2018), that deposited the GSE105269 dataset [29]. Furthermore, hsa-miR-122-5p was found to be significantly upregulated in later stages of the disease for the XFG group compared to earlier phases. In particular, this miRNA is linked to the TGF–β1 pathway (involved in fibrosis and extracellular matrix remodeling) [57]. On the other hand, hsa-miR-320a was found to be upregulated in the AH of XFG patients in comparison with normal subjects [58]. From our re-analysis of the network built with the MIRNET web server (Figure 3), the node with the highest centrality was hsa-let-7a-5p, which was upregulated in the XFG group compared to the CTRL group, according to the GEO2R analysis. Others top nodes in the MIRNET network were hsa-mir-122-5p and hsa-mir-320a, followed by APP, which was also found in the network built on the basis of a POAG vs. CTRL comparison. Additionally, hsa-mir-128-3p, hsa-mir-603 miRNAs, and SUMO2, not previously associated with this disease, were the most central nodes of the XFG vs. CTRL network. A KEGG enrichment analysis with MIRNET (https://www.mirnet.ca/ accessed on 1 March 2022) evidenced that the “Neurotrophin signaling pathway” (linked nodes: BDNF, YWHAQ, and NTRK3); the “p53 signaling pathway”; (linked nodes: TP53, CDK4, CDKN2A, CDKN1A, BAX, and CASP3); and the “Insulin signaling pathway” (linked nodes: MTOR and AKT3) were the most significant signaling pathways of the network. Therefore, this enrichment evidenced pathways that are common to the two different networks representing GSE105269, XFG vs. CTRL, and POAG vs. CTRL. Other interesting significant signaling pathways were the “TGF-beta signaling pathway” (linked nodes: SMAD3 and TGFBR1), the “Wnt signaling pathway”, and the “MAPK signaling pathway”.

Moreover, a rescoring based on entries in PubMed and clinicaltrials.gov evidenced new predicted pharmacological targets for the treatment of XFG: GRK5 (the G protein-coupled receptor kinase 5), GRID1 (the glutamate ionotropic receptor delta type subunit 1), NFKB2 (nuclear factor kappa B subunit 2), PIK3R1 (phosphoinositide-3-kinase regulatory subunit 1), VHL (the von Hippel Lindau tumor suppressor), PKM (pyruvate kinase M1/2), TRPC4 (the transient receptor potential cation channel subfamily C member 4), P2RX3 (the purinergic receptor P2X3), and IGF2R (the insulin-like growth factor 2 receptor). Interestingly, SUMO2, which was identified in the POAG vs. CTRL network, also displayed high closeness centrality values in the XFG vs. CTRL network.

Moreover, 13 genes, already known to be implicated in this disease, were central, according to our network analysis, including the small ubiquitin-like modifier 1 (SUMO1) and IGF1R, which are target genes of the miRNA hsa-let-7a-5p. SUMO1 has been identified as a gene involved in POAG pathogenesis [59], as well as IGF1R [29]. Our analysis also identified the androgen receptor (AR) as a central node, which has been found to be upregulated in glaucomatous ONH astrocytes both in vivo and in vitro [60]. The genes largely described to be involved in glaucoma, which were identified in the XFG vs. CTRL network, include the following: HIF1A (node linked to the enriched “mTOR signaling pathway”); SOD2, which is a target of the central node hsa-let-7a-5p; and TGFBR2, belonging to the “TGF-beta signaling pathway” [61]. Other examples of “non innovative targets” were the nuclear factor NFKB1 and BAX (nodes linked to the “Neurotrophin signaling pathway”, the “p53 signaling pathway”, and “Apoptosis” enriched pathways). Another central node in the XFG vs. CTRL network was VEGFC, which encodes the vascular endothelial growth factor C that was found to be expressed in the trabecular meshwork (TM) and Schlemm’s canal of the endothelial cells of patients with NTG and POAG [62]. Additionally, Hase et al. (2021) showed that the VEGFC expression is induced in TM cells by hypoxia, thereby evidencing the link between VEGFC and HIF1A [62].

### 2.3. GSE27276: Analysis of Differentially Expressed Gene Profiles in Cultured Human Trabecular Meshwork Tissues from Primary Open-Angle Glaucoma Patients Compared to Cells Isolated from Control Healthy Subjects

**Figure 4:**
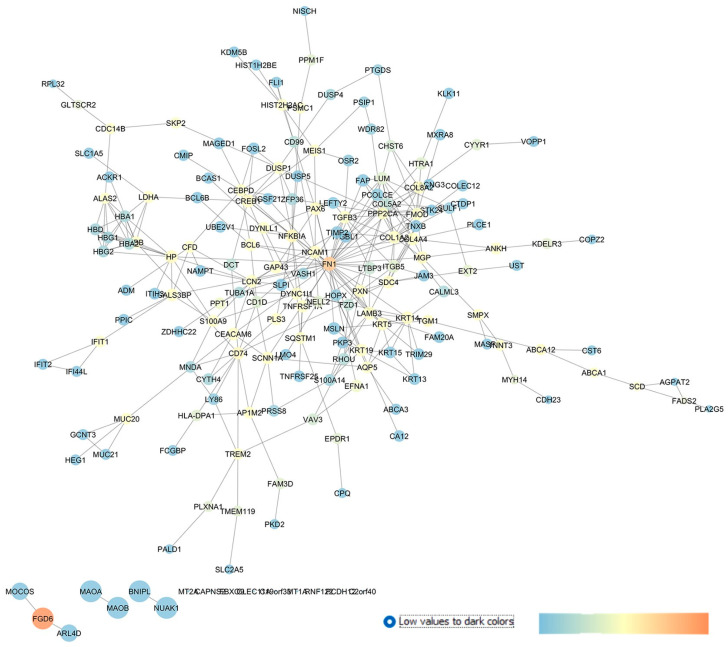
GSE27276 network describing the differences between the expression pattern of cultured trabecular meshwork cells in isolated tissues from POAG patients and healthy subjects. The network has been built with STRING app of Cytoscape, and the centrality parameters are mapped as follows: closeness centrality is proportional to node dimension, betweenness centrality is represented with a temperature color scale, and edge betweenness is proportional to edge thickness.

GSE27276 is a repository containing the gene expression profile of trabecular meshwork (TM) cells isolated from POAG patients compared to cells isolated from the TM of healthy subjects (CTRLs) [30]. Liu et al., by comparing the transcriptome of POAG patients with control subjects, found a list of significant DEGs in POAG, which were mainly associated with endocytic and exosome pathways [30]. With respect to the article by Liu et al. 2013 [30], our re-analysis identified CALML3 and LCN2, included by these authors in the list of top 30 DEGs. The top 250 DEGs retrieved with our GEO2R analysis (POAG vs. CTRL) were used as an input of the STRING app within the Cytoscape environment, and the network in Figure 4 was built.

An analysis of the centrality metrics of this network identified a series of drug targets that can be classified as predicted new pharmacological targets for the treatment of glaucoma, since no entries on PubMed or clinicaltrials.gov were found: NUAK1 (NUAK family kinase 1), MAOB (monoamine oxidase B), HTRA1 (HtrA serine peptidase 1), LDHA (lactate dehydrogenase A), TNFRSF1A (the TNF receptor superfamily member 1A), FZD1 (frizzled class receptor 1), and MTNR1A (the metabotropic melatonin 1A receptor).

Among the in silico identified new drug targets, i.e., genes or proteins not previously linked to glaucoma, we identified the following: SCNN1A (sodium channel epithelial 1 subunit alpha); CACNG3 (calcium voltage-gated channel auxiliary subunit gamma 3); PKD2 (polycystin 2, the transient receptor potential cation channel); SDC4 (syndecan 4, transmembrane heparan sulfate proteoglycan); and HP (haptoglobin).

Our analysis identified several drug targets already linked to glaucoma, strengthening the validity of the reverse engineering approach, hereby used. Fibronectin 1 (FN1) had the highest closeness and betweenness centrality values in our network and was found to be upregulated in immortalized and primary human TM cells through a stimulation of the αvβ3 integrin pathway and contributes to increased TM resistance in glaucoma [63]. As mentioned above, LCN2 (i.e., lipocalin 2) was between the top 30 DEGs identified by Liu et al. (2013), who deposited the GSE objective of this study [30]. LCN2 was also a central gene in our network and encodes for an iron-trafficking protein which plays key roles in innate immunity, apoptosis, and cell proliferation. Moreover, the involvement of lipocalin 2 was confirmed in two in vivo models of glaucoma, spontaneous glaucomatous DBA/2J mice [64], and in a model of mice with an optic nerve crush (OCN) injury [65], respectively. Within the high centrality nodes, GAP43 (the growth-associated protein 43) was found to be involved in retinal ganglion cell death in an experimental chronic glaucomatous injury [66].

DUSP1 also showed elevated betweenness centrality parameters in our network. This gene encodes a nuclear phosphatase (with dual specificity for tyrosine and threonine) and was found to regulate mitogen-activated protein kinase p38 in a retinal ischemic preconditioning [67]. Among the high centrality nodes in our network, AQP5 (aquaporin 5) was recently identified, through exome sequencing, as a new candidate gene for familial cases of POAG [68].

In summary, from an analysis of GSE27276, we found 15 genes with no hits on PubMed and clinicaltrials.gov and 10 genes already known to be involved in glaucoma.

### 2.4. GSE4316: Analysis of Differentially Expressed Gene Profiles in Trabecylar Meshwork Tissues of Primary Open-Angle Glaucoma Patients

This GEO dataset includes the gene expression profiles of the TM tissues of POAG patients [31]. Liton et al. 2006 [31], comparing the gene expression profiles of CTRL and POAG TM tissues (isolated postmortem tissues from patients), found that the top deregulated genes were coding proteins involved in inflammation such as: chemokine (C-X-C motif) ligand 6, chemokine (C-C motif) ligand 5, immune-associated nucleotides, the interleukin 1 receptor type II, transthyretin, haptoglobin, and myelin basic proteins. In addition, Liton et al. 2006 identified the following as upregulated genes in POAG TM tissues: prokineticin 2, the G protein-coupled receptor 146, the regulator of G-protein signaling 1, the neuropeptide Y receptor Y2, and the adenosine A3 receptor [31].

**Figure 5:**
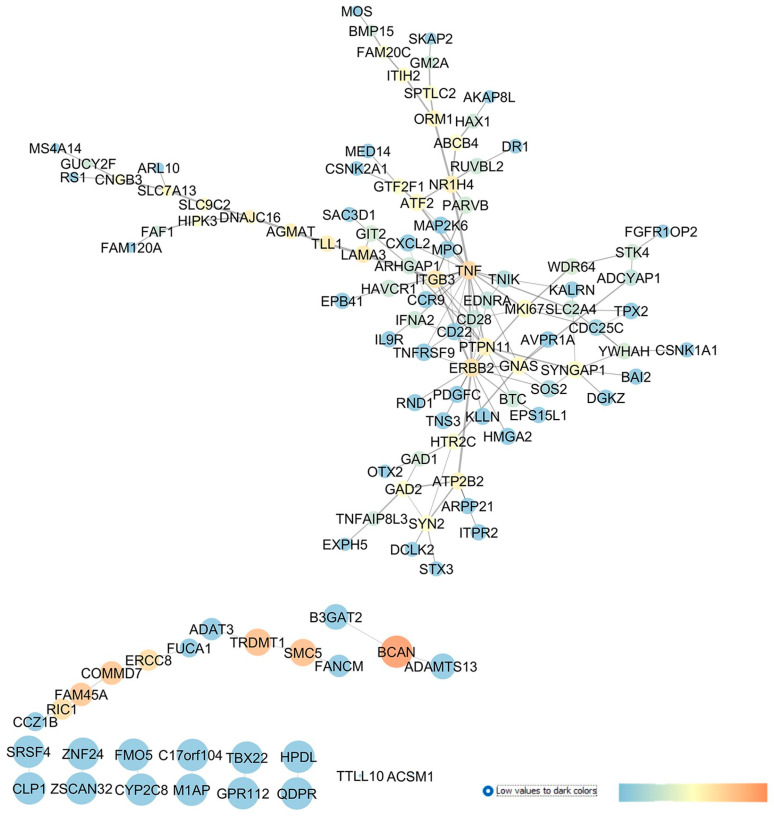
GSE4316 re-analysis and network building, comparing gene expression profile of TM control tissues vs. tissues isolated from POAG patients. In this network, analyzed in Cytoscape, nodes are represented on the basis of betweenness centrality values (color scale blue < red) and closeness centrality values (node dimension), and edge thickness is proportional to edge betweenness values.

According to the methods reported in our study, we carried out a GEO2R statistical analysis, retrieving 250 DEGs in GSE4316, which were the input of the STRING app of Cytoscape, which was used to build the interaction network shown in Figure 5.

Our study highlights several genes and druggable targets which gave no result in the search done on PubMed or on clinicaltrials.gov database; therefore, these may be innovative targets for the treatment of glaucoma: HPDL (4-hydroxyphenylpyruvate dioxygenase like); QDPR (quinoid dihydropteridine reductase); GPR112 (the orphan receptor, G protein-coupled receptor G4); FMO5 (flavin-containing dimethylaniline monooxygenase 5); CYP2C8 (cytochrome P450 monooxygenase); B3GAT2 (beta-1,3-glucuronyltransferase 2); FUCA1 (alpha-L-fucosidase 1); ADAMTS13 (ADAM metallopeptidase); RIC1 (the RIC1 homolog, RAB6A GEF complex partner 1); HTR2C (the 5-hydroxytryptamine receptor 2C); GAD2 (glutamate decarboxylase 2); and HIPK3 (homeodomain-interacting protein kinase 3).

The role of complement system in IOP elevations and then in glaucoma was recently investigated [69]. Interestingly, we found two members of the complement system central to the network describing GSE4316, CD28 and the TNF receptor superfamily member 9, TNFRSF9, but no entries about the involvement of these genes in glaucoma were found. Moreover, ITGB3, which encodes integrin beta-3 (β3), showed an elevated betweenness centrality value in our network, and its role in POAG has not yet been investigated; however, integrin signaling pathways may have a role in the pathogenesis of glaucoma and in the increase in TM resistance to the AH outflow [70].

Our re-analysis of GSE4316 identified several genes already experimentally associated with glaucoma. For example, BCAN (brevican), which is a member of the lectican family of chondroitin sulphate proteoglycans specifically expressed in the central nervous system and known to be released from reactive astrocytes [71], and it showed elevated centrality values in our network. BCAN upregulation was found in rats subjected to ONC injury [72] and in the optic nerve of rats exposed to ischemia/reperfusion (I/Rdamage [73]. Furthermore, TNF, i.e., the tumor necrosis factor, showed elevated betweenness centrality values in the network, and this proinflammatory cytokine has already been proven to have a role in POAG pathophysiology [74].

Moreover, our network centrality analysis identified, as a central node, the gene EDNRA, which encodes the GPCR endothelin receptor type A (ET-A), whose activation triggers vasoconstriction. In the study conducted by Howell et al., in 2014 [75], ET-A was expressed in the ONH vascular endothelial cells of DBA/2J mice, which spontaneously developed with aging glaucoma signs, resembling features in patients. Particularly, the endothelin receptor antagonist bosentan, protected RGCs from neurodegeneration and death [75]. Recently, it has been shown that the retinas of EDNRA-conditioned KO mice in vascular mural cells were protected by endothelin-1 administration, confirming the role of ET-A in glaucomatous neurovascular dysfunctions [76]. Furthermore, a phase I clinical trial was carried out on POAG subjects treated with bosentan (500 mg o.s. and an 8-day treatment) to study the effects of endothelin antagonist treatment in ocular blood flow (NCT00701597). No study results have been published about this trial, but bosentan is currently under evaluation in a randomized controlled trial (NCT02377271) including patients with nonarteritic anterior ischemic optic neuropathy (NAAION) [77].

Finally, YWHAH (tyrosine 3-monooxygenase/tryptophan 5-monooxygenase activation protein eta) was also found to be central in the network, as shown in Figure 5, and it has already been investigated in glaucoma [78].

### 2.5. GSE45570: Analysis of Differentially Expressed Gene Profiles in Optic Nerve Head of Primary Open-Angle Glaucoma Patients and Ocular Hypertensive Patients without Glaucoma

**Figure 6:**
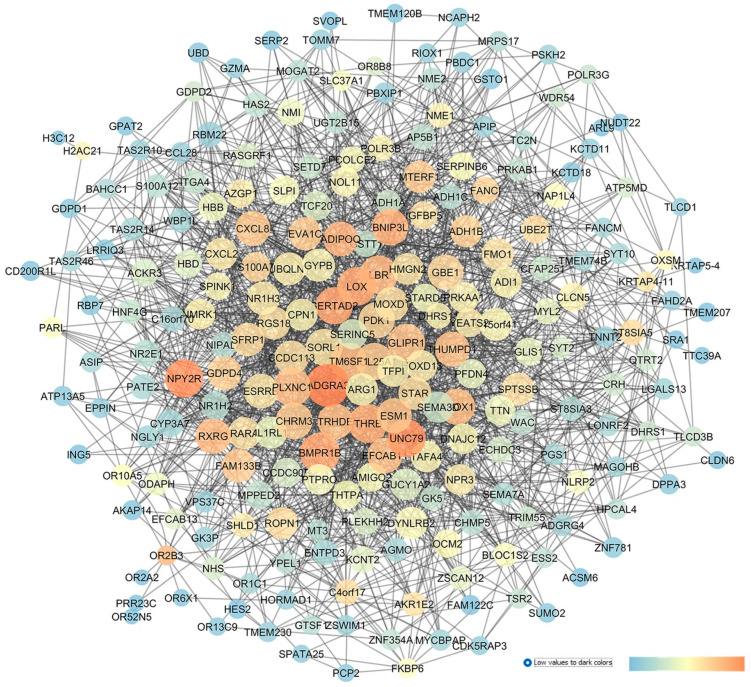
GSE45570 network representing a comparison of the optic nerve head expression profiles of patients with ocular hypertension (without glaucoma) vs. CTRL subjects. This network was analyzed with Cytoscape: the node size is proportional to closeness centrality, the color node represents betweenness values ranging from blue to red (blue to red for increasing values), and edge thickness values are proportional to those of edge betweenness.

GSE45570 includes transcriptomic data from the optic nerve head (ONH) of three experimental groups: patients with primary open-angle glaucoma (POAG), subjects with ocular hypertension (OHT) without glaucoma, and healthy control donors (CTRLs). The aim of our analysis was to discriminate the dysregulated genes involved in IOP elevation (OHT conditions) from genes differentially expressed in the ONH of POAG subjects. In the first comparison, 6 sample groups of ONH from OHT patients and 6 sample groups of ONH from control donors were analyzed with GEO2R. Then, the network in Figure 6 was built, setting as input of the app STRING of Cytoscape the first 250 found DEGs. An analysis of the centrality metrics of the network was carried out, followed by match of genes with PubMed and clinicaltrials.gov entries, which evidenced that 11 genes have not already been associated with glaucoma, meaning that these genes have a high potential to be innovative pharmacological targets for the management of glaucoma: ADGRA3 (the adhesion G protein-coupled receptor A3), NPY2R (the neuropeptide Y receptor Y2), UNC79 (encoding for an auxiliary subunit of the NALCN sodium channel), BMPR1B (the bone morphogenetic protein receptor type 1B), OR2B3 (the olfactory receptor family 2 subfamily B member 3), THRB (the thyroid hormone receptor beta), RXRG (retinoid x receptor gamma), LBR (the lamin B receptor), ADIPOQ (adipokine), BBOX1 (gamma-butyrobetaine hydroxylase 1), and TRHDE (the thyrotropin-releasing hormone-degrading enzyme).

Furthermore, the network describing GSE45570 (OHT vs. CTRL) identified other genes with high closeness or betweenness centrality values; however, these were already found to be involved in glaucoma. Among these, LOX (lysyl oxidase) showed high network centrality parameters; this gene encodes a copper-dependent enzyme that regulates the oxidative deamination of the lysine residues of elastin and collagen. LOX activity has been found to increase trabecular meshwork stiffness, thereby increasing the outflow of the aqueous humor and then IOP [79]. Our network analysis identified the gene CHRM3 (the cholinergic muscarinic receptor 3, M_3_R) which has high betweenness or centrality values, and this receptor has already been studied as an intriguing pharmacological target for glaucoma treatment. In fact, the acetylcholinesterase inhibitor, huperzine A, is able to decrease IOP in New Zealand rabbits, through the indirect activation of M_3_R [80].

**Figure 7:**
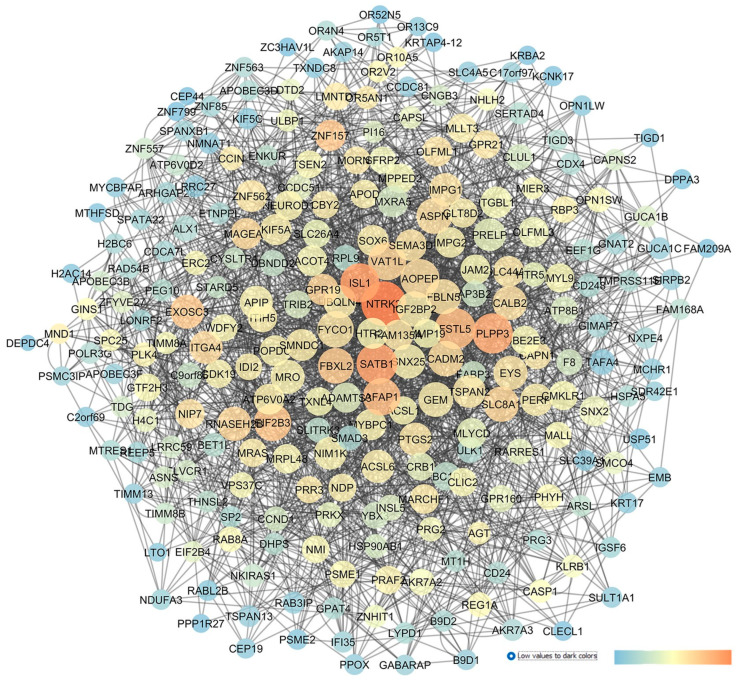
GSE45570 network describing a comparison of the optic nerve head expression profiles of patients with POAG vs. CTRL subjects. This network was analyzed with Cytoscape: the node size is proportional to closeness centrality, the color node represents betweenness values ranging from blue (low betweenness values) to red (high betweenness values), and edge thickness values are proportional to those of edge betweenness.

The re-analysis of GSE45570, comparing the following samples, POAG (6 samples) vs. CTRL (6 samples), was conducted, which led to the network shown in Figure 7. The network centrality analysis evidenced the following five central nodes as the new predicted genes involved in glaucoma, that can be potentially further validated for glaucoma treatment, given that no entries were retrieved about these genes on PubMed and clinicaltrials.gov: HTR5A (5-HT5A), FSTL5 (follistatin-like 5), EXOSC3, (exosome component 3), FBXL2 (the f-box and leucine-rich repeat protein 2), and CLIC2 (the chloride intracellular channel 2).

Other central nodes in this network have already been associated with glaucoma, such as NTRK2 (the neurotrophic receptor tyrosine kinase 2, alias TRKB), which showed the highest centrality values inside the network. TRKB is activated by the brain-derived neurotrophic factor (BDNF), and the BDNF–TRKB signaling pathway has already been proven to stimulate the survival of RGCs in different models of retinal injuries [81,82]. PLPP3 (phospholipid phosphatase 3) showed high centrality metric parameters, and it was found to be significantly overexpressed in the optic nerve of glaucoma patients [83]. With respect to AFAP1 (the actin filament-associated protein 1), implicated in the cross-linking of actin filaments, this gene has been associated with elevated IOP values or to increase POAG risk factor, as evidenced by several gene-wide association studies (GWASs) [84,85]. Among the nodes with moderate centrality values, the HTR2A gene (the 5-HT2A receptor) has already been linked to glaucoma. Particularly, a treatment using 5-HT2A agonists decreased the IOP in Cynomolgus monkeys [86]. Furthermore, cabergoline (a dopaminergic and serotoninergic agonist) decreased the IOP in a mouse model of steroid-induced ocular hypertension [87]. However, up to now, the exact mechanism of the serotonergic system in modulation of IOP levels has not been fully elucidated.

**Figure 8:**
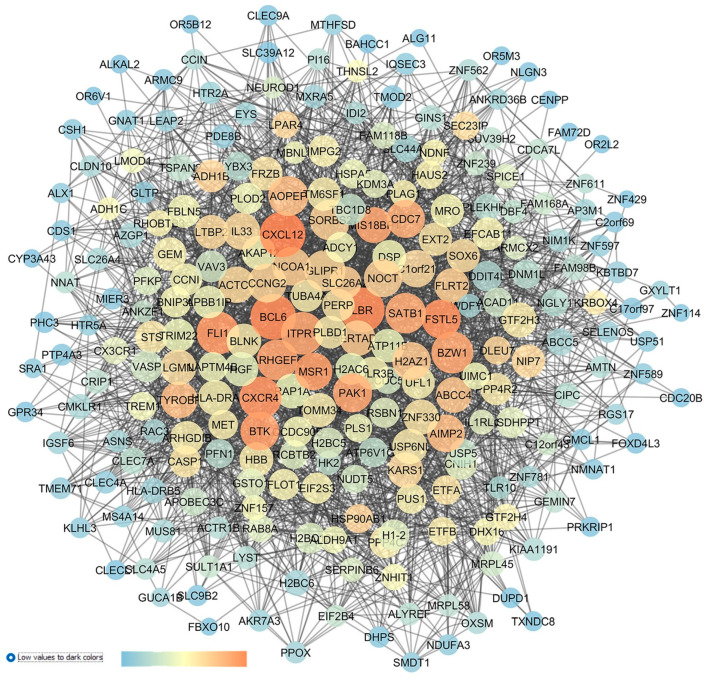
GSE45570 network representing the differences of the optic nerve head expression profiles of patients with POAG vs. patients with ocular hypertension (without glaucoma). This network was analyzed with Cytoscape: the node size is proportional to closeness centrality; the color node represents betweenness values, ranging from a blue to red of temperature color scale (shown in figure), with increasing values going from blue to red; and the edge thickness values are proportional to those of edge betweenness.

Finally, with respect to GSE45570, the GEO2R analysis was carried out comparing 6 samples from POAG patients with 6 samples of OHT patients. This comparison was carried out in order to highlight the DEGs putatively involved in ocular hypertension progression to POAG, or to at least differentiate between these two conditions. The top 250 DEGs were set as input of the STRING app within Cytoscape, and the enriched network (Figure 8) was built.

The centrality analysis of the network highlighted (high centrality values) 8 genes as new in silico identified dysregulated genes in OHT and POAG, with no entries in the literature or in clinical trials: LBR (the laminin B receptor, already identified in the OHT vs. CTRL network); FSTL5 (follistatin-like 5, already identified in the POAG vs. CTRL network); IL3 (interleukin 3); BTK (bruton tyrosine kinase); MSR1 (the macrophage scavenger receptor 1); ARHGEF6 (the Rac/Cdc42 guanine nucleotide exchange factor 6); PAK1 (serine/threonine p21-activating kinases); and MIS18BP1 (the MIS18-binding protein 1).

Other nodes central in this network have been also identified in the re-analysis of a comparison of POAG vs. OHT in GSE45570, and these nodes have already been linked to glaucomatous condition. Specifically, we again identified the gene HTR2A (serotonergic 5-HT2A receptor) [86,87]. Furthermore, CXCL12 (C-X-C motif chemokine ligand 12) was the most central node in the network, and it was found that through an activation of the CXCR3 receptor, this chemokine promotes caspase activation, leading to apoptosis, and IOP elevation in a rat model of ocular hypertension [88]. Another chemokine, the node CXCR4 (C-X-C motif chemokine receptor 4), was found to be central in the network (Figure 8), and its expression influenced the trabecular meshwork morphology of POAG patients [88,89,90]. ITPR1 (the inositol 1,4,5-trisphosphate receptor, type 1) also displayed elevated closeness centrality values in the network, and it was found to be dysregulated in the AH of POAG patients [91]. Finally, CDC7 (cell division cycle 7) was identified as a central node in our analysis, and polymorphisms of CDC7/TGFBR3 have been associated with glaucoma [92], along with visual field progression and a reduction in the thickness of the retinal nerve fiber layer of glaucoma patients [92,93,94].

### 2.6. GSE3554: Analysis of DBA/2J Mice Retinal Gene Expressions

The GSE3554 reports the expression profiles of the retinas of 3-month- and 8-month-old DBA/2J mice [32]. These DBA/2J mice spontaneously developed glaucoma, and at 8 months, the IOP significantly increase, and the mice also display retinal dysfunction and an altered cell morphology [95]. Steele et al., 2006 [32], validated, with a quantitative RT-PCR the microarray results for ceruloplasmin, chitinase 3-like 1, lipocalin 2, the complement component 1q, chemokine ligand 12, and the interferon-induced transmembrane protein 1 [32]. We were also able to identify, with our informatic analysis, lipocalin 2 (GSE27276) and chemokine ligand 12 (GSE45570: POAG vs. OHT). We applied to GSE3554 the bioinformatic protocol hereby presented, but we were not able to identify any central node in the network, because we did not found any centrality or topological parameter able to discriminate nodes in the network.

### 2.7. Integration with Preclinical and Clinical Data

**Figure 9:**
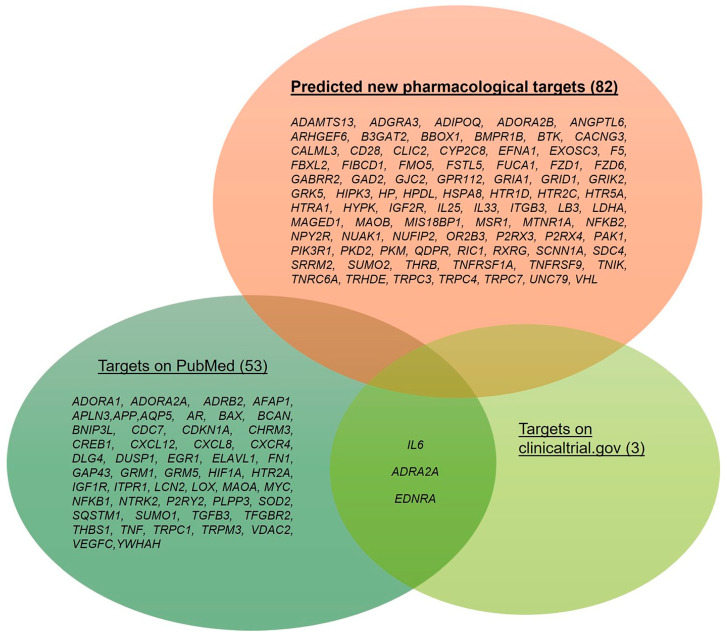
Set of dysregulated genes identified after our re-analyses of the GSE133563, GSE105269, GSE27276, GSE4316, GSE45570, and GSE3554 datasets. Genes that were already evaluated in clinical trials or preclinical studies are distinguished from those without a match in the PubMed and clinicaltrials.gov databases, i.e., predicted new pharmacological targets. The number of genes is shown in brackets.

After the network analysis of the different expression datasets and identification of the central genes in the network, we matched results with entries from the PubMed (https://pubmed.ncbi.nlm.nih.gov/ accessed on 1 October 2022) and/or clinicaltrials.gov (https://clinicaltrials.gov/ accessed on 1 October 2022) databases. The genes, without hits related to literature studies or clinical trials, can be considered as new predicted pharmacological targets for the treatment of glaucoma and would need an in vitro and in vivo validation. Overall, we identified 135 genes over the six analyzed GEO datasets. A total of 82 out of 135 had not match within PubMed and clinicaltrials.gov database. Figure 9 shows the whole set of genes identified after an analysis and rescoring of the GSE dataset networks, built de novo through our in silico approach. It is worth noting that genes encoding for GPCRs and ligand-gated ionic channels and enzymes (easily druggable targets) were in the subset of “new pharmacological targets”. Clinical studies were identified only for three genes out of 135 identified central nodes: IL6, ADRA2A, and EDNRA. This result would highlight the current attrition in the clinical development of new drugs or interventions for glaucoma management.

## 3. Discussion

In this work, six GEO datasets were analyzed. These datasets report transcriptomic analyses of tissues primarily implicated in the etiopathogenesis of glaucoma (trabecular meshwork, optic nerve head, whole retinal samples, retinal ganglion cells, and aqueous humor) and isolated from in vivo models of diseases or isolated from patients. A bioinformatic analysis of these datasets was carried out, using a system biology or system pharmacology approach, with the aim of identifying genes and miRNAs that may have a role in glaucoma pathogenesis. As was already found in a previous study published by our group [15], this approach is unbiased, and a main strength of this methodology is the rescoring of nodes central in the networks through a match with the PubMed and clinicaltrials.gov databases. In fact, we were able to identify genes that were already found to have an experimental link to glaucoma. Specifically, genes have been linked, through a gene ontology analysis or directly through a network analysis, to inflammatory, immune system, oxidative stress, and fibrotic pathways, which have already been associated with mechanisms involved in glaucoma [26,27,96,97,98].

For instance, several inflammatory cytokines and chemokines, i.e., IL-6, TNF, CXCL12, and CXCL8, showed elevated centrality values in various networks, evidencing the detrimental role of neuroinflammation in glaucoma [99]. Several studies have found an overexpression of these pro-inflammatory cytokines in retinal glaucomatous tissues and AH samples [100,101]. Moreover, elevated levels of the pro-inflammatory cytokine CXCL8 were detected in the AH of POAG, XFG, and neovascular glaucoma (NVG) subjects and were also associated with an elevated preoperative IOP or visual field defects in the eyes of patients [100]. Furthermore, an upregulation of TNF-α was found in the AH of POAG and XFG eyes [102]. Interestingly, TNF-α and the TNF-α receptor 1 were found to be upregulated in retinal glial cells and RGCs postmortem in the eyes of POAG donors [103]. Accordingly, different genes related to the TNF-*α* signaling pathway, such as TNFRSF1A and TNFRSF9, were enriched in our networks, strengthening the involvement of immunopathological mechanisms in glaucomatous retinas [74].Additionally, several components related to the complement cascade, such as CD28 and TNFRSF9, were identified in our in silico analysis, in accordance with evidence from other already published works [104,105]. Our study also highlighted the involvement of the NFKB1 and NFKB2 genes, that encode transcription factors regulating innate immune system-related responses. In this regard, Reinehr et al., in 2018 [106], found an increase in NFkB in S100 retinas (autoimmune glaucoma model). Furthermore, we identified the gene encoding HIF-1α as a crucial gene in our network analyses, and several experimental reports, along with one on the etiopathogenic hypothesis of the onset and progression of glaucoma (i.e., perfusion deficits at the optic nerve head), corroborated the detrimental role of this transcription factor in glaucoma pathogenesis [36,37,38]. Interestingly, HIF-1α was also found to be upregulated in an inherited glaucoma model of DBA/2J mice and, along with its target gene VEGF, was suggested to participate to in the breakdown of the blood–retinal barrier, thus promoting the loss of RGCs [107]. In this regard, our in silico analysis also identified VEGFC, which was found to be upregulated in the trabecular meshwork of patients with POAG and neovascular glaucoma [62]. Specifically, the VEGF pathway is already known to promote neovascularization in different retinal neurodegenerative diseases, as well as in glaucoma [108]. Additionally, our analysis identified different glutamate receptors in the de novo built and analyzed networks, confirming the experimental finding regarding the role of glutamate excitotoxicity as a detrimental factor in glaucoma neurodegeneration. In detail, GRM1, encoding for mGlur1, was identified as a central node in the network describing GSE133563. The signaling of mGluR1 has already been described in the degeneration of RGCs by Liberatore et al., 2017 [109], where the excitotoxic damage of RGCs, induced by a toxic dose of monosodium glutamate in mice, was reversed by treating the animals with an mGluR1-negative allosteric modulator [109]. Furthermore, mGluR1 also has a role in the activation of Müller cell gliosis [110,111]

With respect to apoptotic events that lead to the programmed death of RGCs, our in silico analysis identified several genes central in the networks, such as BAX (identified as “GSE105269 XFG vs. CTRL” in the network analyses) and the “p53 signaling pathway” (enriched in the MIRNET GSE105269 networks). Furthermore, there is strong evidence to support the claim that apoptosis is linked to neurotrophin deprivation [112]. Indeed, our in silico analysis identified the genes involved in neurotrophin signaling, such as NTRK2, i.e., the tyrosine kinase receptor of BDNF is central gene in the network describing the GSE45570 network “POAG vs. CTRL”.

Our bioinformatic analysis identified the gene ELAVL1 as a central node in the networks, and this chaperonine is a key regulator of gene expressions at the post-transcriptional level, which was recently found to be fundamental for the survival and protection of RGCs [113]. In the work by Smedowski et al., (2018) [42], ELAVL1 was detected in the retinas and optic nerves of glaucomatous rats, with an altered nucleus and cytoplasmic localization. Additionally, after the bioinformatic re-analysis of GS105269, we identified, as central node, APP. These results strengthen the validity of our in silico method, since there are several pieces of evidence supporting the common pathogenesis of glaucoma and Alzheimer’s diseases [44,45,114].

Furthermore, our bioinformatic analysis was able to identify several druggable targets for glaucoma treatment, e.g., the A_1_ and A_2A_ adenosine receptors, along with the 5-hydroxytryptamine 2A and purinergic P2Y2 receptors, which have already been investigated in other studies. Indeed, adenosine receptors (identified in the GSE133563 network analysis) have already been hypothesized to be useful for the treatment of glaucoma, due to their recognized role in controlling the IOP [115]. More specifically, a blockade of the microglial adenosine A2A receptor proved to be protective against a retinal dysfunction triggered by an elevation of the IOP [116], while an activation of the adenosine A1 receptor demonstrated to reduce the IOP by increasing the outflow facility in mice [117]. Additionally, caffeine, a non-selective adenosine receptor antagonist with a higher affinity for the adenosine A_1_ and A_2A_ receptors, showed retinal protective effects in a mouse model of I/R injuries, which resembled glaucomatous neurodegeneration [118]. Furthermore, the serotoninergic system and sigma receptor have already been investigated for their role in modulating the IOP and neurodegeneration [86,87,119]. Additionally, the purinergic receptor P2Y2, identified by the GSE105269 network analysis, was previously found to be a potential dysregulated receptor in glaucoma. Specifically, P2RY2 receptor agonists were found to increase the IOP [50]. Furthermore, Fonseca et al., in 2017 [51], found an overexpression of P2RY2 in DBA/2J mice, strengthening the involvement of this receptor in the onset and progression of glaucoma [51].

Rescoring on the basis of matching nodes in the built networks with entries in the PubMed and clinicaltrials.gov databases was necessary in order to identify the genes, and then related proteins, that can be considered innovative targets for the treatment of glaucoma. For instance, 82 out of 135 identified targets have not yet been experimentally associated with glaucoma. For instance, we characterized these in silico identified nodes in networks as predicted new pharmacological targets for the treatment of glaucoma. A main limitation of our study is related to the in silico approach, which is merely a predictive approach, given that these targets need experimental validation. One strength of the reverse engineering approach hereby used is related to the methods of in silico system biology approaches, namely, these are successful in their description of the pathogenic mechanisms of complex multifactorial diseases, facilitating the R&D process of drug development [120]. Several GPCRs were classified, through rescoring, as predicted new pharmacological targets: the adenosine A_2B_ receptor, the 5-hydroxytryptamine receptor 1D, the 5-hydroxytryptamine receptor 2C, the 5-hydroxytryptamine receptor 5A, the melatonin receptor 1A, and the olfactory receptor family 2 subfamily B member 3. Moreover, among the innovative druggable targets, we found the following: the purinergic ionotropic receptors P2X3 and P2X4; the chloride intracellular channel 2 (CLIC2); and the cation channels TRPC3, TRPC4, and TRPC7. Interestingly, the role of the purinergic system in glaucoma has already been described for the P2X7 receptor [121,122]. Moreover, different subunits of glutamatergic receptors, e.g., GRIA1 and GRIK2, encoding the glutamate ionotropic receptor AMPA type subunit 1 and the glutamate ionotropic receptor kainate type subunit 2, respectively, identified as central nodes in the GSE133563 network, have not yet been associated with glaucoma pathogenesis. We were also able to identify, as intriguing and innovative targets, genes encoding for enzymes: CYP2C8, LDHA, MAOB, and B3GAT2.

## 4. Materials and Methods

### 4.1. GEO Dataset

The Gene Expression Omnibus Dataset repository (GEO dataset https://www.ncbi.nlm.nih.gov/gds accessed on 1 March 2022) [22] was the database from which the datasets listed in Table 1 were retrieved, by the means of the keyword “glaucoma”. DEGs (*p* < 0.05) for each sample of the GEO datasets (e.g., glaucoma vs. healthy control) were identified using the GEO2R statistical analysis (https://www.ncbi.nlm.nih.gov/geo/info/geo2r.html accessed on 1 March 2022), applying the Benjamini and Hochberg (false discovery rate) method for the adjustment of *p*-values using standard parameters in GEO2R [23,123,124]. In each GEO dataset comparison, approximately 250 DEGs with a *p*-value of <0.05 were then selected as an input of the STRING app within Cytoscape.

### 4.2. Network Definition and Descriptor Computations

STRING (© STRING CONSORTIUM 2018), applied with Cytoscape v. 3.7.0 (2001–2017 Cytoscape Consortium), was employed to carry out an enrichment of information and to build gene networks (https://apps.cytoscape.org/apps/stringapp, accessed on 1 March 2022) [25]. After the entry of a list of genes, setting a confidence score cut-off (0.40) and the maximal number of additional interactors as 20, the STRING app, by using a wide dataset of direct (physical) as well as indirect (functional) association [125], provided the annotated Cytoscape networks based on the predicted integration of protein–protein interactions. The enrichment data were loaded. A network analysis was carried out through Cytoscape, and all networks were treated as unweighted and undirected graphs. The adjacency matrix A is the mathematical representation of the network:

$$A_{ij}=\left\{\begin{array}{c} 1\;if\;the\;residue\;i\;and\;j\;are\;in\;contact\\ 0\;\;\;\;\;\;\;\;\;\;\;\;\;\;\;\;\;\;\;\;\;\;\;otherwise\;\;\;\;\;\;\;\;\;\;\;\;\;\;\;\;\;\;\;\;\;\;\;\;\;\;\;\;\; \end{array}\right.$$

On the basis of the definition of matrix A, a series of network descriptors, representing the topological role of nodes (genes) in the networks, could be calculated. Node degree $k_{i}$ is the network primary descriptor: it defines the number of nodes with which every node is in contact. The shortest path between two nodes is determined as the minimum number of links connecting the two nodes. Based on the shortest paths, two centrality metrics, i.e., closeness centrality and betweenness centrality, could be defined.

Closeness centrality depends on the sum of the minimal distance of one vertex (*V*) to all other vertices, considering the length of the shortest paths connecting them. In particular, the closeness centrality (close) of the *i*-th node can be mathematically calculated as follows:

$$close\left(i\right)=\frac{1}{\sum_{u\epsilon V,u\neq i}{sp}_{ui}}$$

Betweenness centrality quantifies the times in which one vertex acts as a bridge between two other vertices. Specifically, given a set of vertices (*V*), the mathematical representation of the betweenness (betw) centrality of the *i*-th node is follows:

$$betw\left(i\right)=\sum_{v\epsilon V,v\neq i}\sum_{u\epsilon V,u\epsilon i}\frac{\sigma_{uv}(i)}{\sigma_{uv}}$$

where $\sigma_{uv}$ is the total number of shortest paths linking nodes *u* and *v*, and $\sigma_{uv}\;(i)$ is the number of shortest paths linking the two nodes and passing through the node *i*.

An analysis of centrality metrics is necessary for the identification of genes that would play a key role in the stability and modularity of a network [126]. Specifically, nodes were represented with a size proportional to closeness centrality values. Betweenness values were plotted by assigning a node a color according to a temperature color scale, which changed from blue to red according to the increase values of the node’s betweenness values. Moreover, edge betweenness was set as proportional as to edge thickness, and the prefuse force layout of the networks was represented on the basis of edge betweenness. Furthermore, in the analysis, the bioinformatic tool MIRNET (https://www.mirnet.ca/ accessed on 1 March 2022) was employed [127].

Finally, after identifying the nodes that were central in the built networks, we matched the nodes with entries in PubMed and clinicaltrials.gov. This rescoring approach was necessary to discriminating nodes (genes or miRNA) that have already been studied in preclinical models of this disease or in glaucoma subjects recruited in clinical trials. Specifically, to match the in silico identified targets with experimental studies (preclinical and/or clinical), the query “target name AND glaucoma” was used to mine data in the PubMed and clinicaltrials.gov databases.

## 5. Conclusions

In conclusion, we identified, through an in silico approach, pharmacological targets that can be considered predicted and new for the potential treatment or management of glaucoma, due to their lack of a match with published data (PubMed) or clinical trials (clinicaltrials.gov). One step forward would be to identify the potential ligands, agomirs, and antagomirs, along with transcription factors or suppressors, of these 82 predicted new targets, i.e., through access to enrichment approaches and related databases: STITCH (http://stitch.embl.de/) [128], DIANA tools (https://diana.e-ce.uth.gr/home) [129], and GeneCards.org (https://www.genecards.org/) [130]. This is the first theoretical step toward the validation of pharmacological targets that can reveal novel treatments of glaucoma, and the most time-consuming and expensive steps are experimental validation of these targets through preclinical and clinical studies. Since the assumed network pharmacology approach, which borrows in silico system biology methods, is unbiased, it can facilitate an R&D process analyzing a complex multifactorial disease through a holistic perspective instead of a reductionist approach. It is worth noting that most of the identified “predicted new” targets are GPCRs, channels, and enzymes; therefore, drug repurposing programs could further facilitate clinical research in glaucoma, hopefully with a focus on neuroprotective strategies.

## Figures and Tables

**Table 1:** Gene Expression Omnibus datasets regarding preclinical (in vivo) models of glaucoma and postmortem isolated tissues from donor glaucoma subjects. POAG = primary open-angle glaucoma; XFG = exfoliation glaucoma; OHT = ocular hypertension; IOP = intraocular pressure.

GEO Dataset Code	Reference	Sample Group 1	Sample Group 2
GSE133563	[28]	Optic nerve crush tissue (retina) of 8-week-old Lister Hooded rats	Sham-operated tissue (retina) of 8-week-old Lister Hooded rats
GSE105269	[29]	Aqueous humor of POAG or XFG patients	Aqueous humor of control patients
GSE27276	[30]	Trabecular meshwork cell culture, whose cells were isolated from POAG patients	Trabecular meshwork cell culture, whose cells were isolated from control subjects
GSE4316	[31]	POAG human trabecular meshwork tissues	Human trabecular meshwork tissues of control donors
GSE45570		Optic nerve head of POAG or OHT patients	Optic nerve head of control subjects
GSE3554	[32]	Tissues (retina) of 8-month-old DBA/2J mice of (with an elevated IOP)	Tissues (retina) of DBA/2J 3-month-old mice (with a normal IOP)

**Table 2:** Central genes in the network built fromGSE105269 re-analysis (POAG vs. CTRL). Closeness centrality depends on the sum of shortest paths between all nodes. The stress centrality of a node measures the number of shortest paths passing through that node, while degree represents the number of nodes it is in contact with.

Node	Closeness Centrality	Stress	Degree
Hsa-let-7b-5p	0.62	2,455,969,486	4489
Elavl1	0.51	123,020,754	1244
Myc	0.49	18,006,672	512
Parp1	0.49	10,943,968	122

## Data Availability

All data generated or analyzed in this study are included in this published article.

## Supplementary Materials

The following supporting information can be downloaded at: https://www.mdpi.com/article/10.3390/ph17101333/s1, List S1: List of the differentially expressed miRNAs input on MIRNET for the analysis of GSE105269 POAG vs. CTRL; List S2: List of the differentially expressed miRNAs input on MIRNET for the analysis of GSE105269 XFG vs. CTRL.

## Abbreviations

ACG	Angle-closure glaucoma
AD	Alzheimer’s disease
AH	Aqueous humour
CTRL	Control
DE	Differentially expressed
DEGs	Differentially expressed genes
ECM	Extracellular matrix
EVC	Episcleral vein cauterization
GPCRs	G protein-coupled receptors
HTM	Human trabecular meshwork
IOP	Intraocular pressure
I/R	Ischemia/reperfusion
KEGG	Kyoto encyclopedia of genes and genomes
LGN	Lateral geniculate nucleus
NF-kB	Nuclear factor kappa-B
NTG	Normal-tension glaucoma
NVG	Neovascular glaucoma
OHT	Ocular hypertension
ONC	Optic nerve crush
ONH	Optic nerve head
POAG	Primary open-angle glaucoma
RGCs	Retinal ganglion cells
TM	Trabecular meshwork
VEGF	Vascular endothelial growth factor
WGS	Whole-genome sequencing
XFG	Exfoliation glaucoma
XFS	Exfoliation syndrome
