# Supplementary material for: The Identification of New Pharmacological Targets for the Treatment of Glaucoma: A Network Pharmacology Approach
Source: Pharmaceuticals (Basel). 2024 Oct 5;17(10):1333. doi: 10.3390/ph17101333 (PMC11509888; doi:10.3390/ph17101333)
Supplement: Supplementary file 1 [file pharmaceuticals-17-01333-s001.zip › pharmaceuticals-3139550-supplementary.pdf]

SUPPLEMENTARY MATERIAL

Identification of novel pharmacological targets for treatment of glaucoma: a network pharmacology approach

Erika Giuffrida[1,†], Chiara B.M. Platania[1,2,†], Francesca Lazzara[1], Federica Conti[1], Nicoletta Marcantonio[1], Filippo Drago[1,2] and Claudio Bucolo[1,2,*]

1 Department of Biomedical and Biotechnological Sciences, School of Medicine, University of Catania, Catania, Italy; erika.giuffri@gmail.com; chiara.platania@unict.it; francesca.lazzara@unict.it; federica.conti@unict.it; nicolettamarcantonio61@gmail.com; fdrago@unict.it; claudio.bucolo@unict.it

2 Center for Research in Ocular Pharmacology-CERFO, University of Catania, Catania, Italy

† These authors equally contributed to the study

* Correspondence: claudio.bucolo@unict.it

List of the differentially expressed miRNAs input on MIRNET for the analysis of GSE105269 POAG vs. CTRL:

hsa-miR-517c-3p

hsa-miR-339-5p

hsa-miR-375

hsa-miR-125b-5p

hsa-miR-520a-5p

hsa-miR-648

hsa-miR-1302

hsa-miR-520h

hsa-miR-21-5p

hsa-miR-3190-3p

hsa-miR-329-5p

hsa-miR-616-3p

hsa-miR-1244

hsa-miR-370-5p

hsa-miR-330-5p

hsa-miR-511-5p

hsa-miR-431-5p

hsa-miR-378g

hsa-miR-302b-3p

hsa-miR-194-5p

hsa-let-7b-5p

hsa-miR-204-5p

hsa-miR-193a-3p

hsa-miR-3195

hsa-miR-513a-3p

hsa-miR-148a-3p

hsa-miR-496

hsa-miR-484

hsa-miR-320e

List of the differentially expressed miRNAs input on MIRNET for the analysis of GSE105269 XFG vs. CTRL

hsa-miR-548ar-5p

hsa-miR-612

hsa-miR-122-5p

hsa-miR-320a

hsa-let-7a-5p

hsa-miR-1253

hsa-miR-128-3p

hsa-miR-1283

hsa-miR-378e

hsa-miR-502-5p

hsa-miR-548aa

hsa-miR-570-3p

hsa-miR-579-3p

hsa-miR-603

hsa-miR-627-5p

hsa-miR-33a-5p

hsa-miR-320e

hsa-miR-610

hsa-miR-758-5p

hsa-miR-3144-3p

hsa-miR-148a-3p

hsa-miR-143-3p

hsa-miR-494-3p

hsa-miR-3190-3p

hsa-miR-1286

hsa-miR-576-5p

hsa-miR-342-3p

hsa-miR-1-5p

hsa-miR-574-5p
